# Human papillomavirus and genome instability: from productive infection to cancer

**DOI:** 10.6061/clinics/2018/e539s

**Published:** 2018-08-28

**Authors:** Bruna Prati, Bruna Marangoni, Enrique Boccardo

**Affiliations:** Departamento de Microbiologia, Instituto de Ciencias Biomedicas, Universidade de Sao Paulo, Sao Paulo, SP, BR

**Keywords:** HPV, Genomic Instability, E6, E7, Cervical Cancer, Synthetic Lethality

## Abstract

Infection with high oncogenic risk human papillomavirus types is the etiological factor of cervical cancer and a major cause of other epithelial malignancies, including vulvar, vaginal, anal, penile and head and neck carcinomas. These agents affect epithelial homeostasis through the expression of specific proteins that deregulate important cellular signaling pathways to achieve efficient viral replication. Among the major targets of viral proteins are components of the DNA damage detection and repair machinery. The activation of many of these cellular factors is critical to process viral genome replication intermediates and, consequently, to sustain faithful viral progeny production. In addition to the important role of cellular DNA repair machinery in the infective human papillomavirus cycle, alterations in the expression and activity of many of its components are observed in human papillomavirus-related tumors. Several studies from different laboratories have reported the impact of the expression of human papillomavirus oncogenes, mainly E6 and E7, on proteins in almost all the main cellular DNA repair mechanisms. This has direct consequences on cellular transformation since it causes the accumulation of point mutations, insertions and deletions of short nucleotide stretches, as well as numerical and structural chromosomal alterations characteristic of tumor cells. On the other hand, it is clear that human papillomavirus-transformed cells depend on the preservation of a basal cellular DNA repair activity level to maintain tumor cell viability. In this review, we summarize the data concerning the effect of human papillomavirus infection on DNA repair mechanisms. In addition, we discuss the potential of exploiting human papillomavirus-transformed cell dependency on DNA repair pathways as effective antitumoral therapies.

## Human papillomaviruses and cancer

Human papillomaviruses (HPVs) are small, nonenveloped viruses with a genome composed of double-stranded circular DNA of approximately 8 kbp 1,2. HPVs belong to the *Papillomaviridae* family and, to date, over 200 different types have been identified and their genomes completely sequenced 1. The HPV genome can be divided in three functional regions. The upstream regulatory region (URR) or long control region (LCR) is a noncoding portion encompassing almost an eighth of the viral genome and is involved in the regulation of viral replication and transcription. In addition, there are two coding regions. The early (E) region harbors the E1, E2, E4, E5, E6 and E7 early genes that are critical for regulating viral genome replication, viral gene expression, immune evasion and genome persistence. The late (L) region encodes the major and minor capsid proteins from the L1 and L2 genes, respectively. These viruses display a marked tropism for stratified epithelial tissues and are transmitted by direct contact with infected skin and mucosa. As such, HPV infections are associated with a variety of benign hyperproliferative conditions, such as common and genital warts.

Approximately 40 HPV types infect the mucosa and skin of the anogenital tract. These HPV types are further subclassified as low or high risk according to their association with the development of cervical carcinoma and its precursor lesions. Low-risk HPV types, such as HPV6 and HPV11, are associated with genital warts and low-grade cervical intraepithelial neoplasia (CIN) 3,4. On the other hand, high-risk HPV types, for example, HPV16, HPV18, HPV31 and HPV45, are associated with the development of high-grade CIN and cervical cancer. In addition, they are associated with a significant proportion of vulvar, vaginal, anal, penile and head and neck carcinomas 5.

To establish a productive infection, HPVs have to reach cells from the basal layer of the epithelium. These are the only keratinocytes of the tissue that proliferate and express the DNA replication machinery. Therefore, they provide a permissive environment for initial viral genome replication. The virus can reach these cells through microwounds caused during sexual intercourse or at specific anatomical sites were the basal layer is readily accessible, such as the squamocolumnar junction (SCJ) of the cervix 6. Once in the cell nucleus, the HPV genome remains in the episomal state (100-500 copies/per cell) and replicates together with the cellular genome 7. The rest of the viral cycle, including vegetative genome amplification, structural protein expression, virion mounting and release, requires infected keratinocytes to migrate through the different layers of the epithelium and complete their differentiation program 8.

In high-grade CIN and HPV-associated tumors, viral DNA is frequently integrated into the host cell genome. Viral integration is an accidental event that usually disrupts the region were the E1 and E2 genes are located, leading to the loss of their expression. This constitutes a critical step in HPV-associated carcinogenesis since the loss of E2 leads to the upregulated expression of the main viral oncoproteins, namely, E6 and E7 9. The sustained expression of E6 and E7 ensures the existence of a cellular milieu favorable for HPV replication in the suprabasal layers of the epithelium and is critical in the process of HPV-mediated cell transformation (see below) 10.

It is estimated that approximately 291 million women are infected by genital HPV types worldwide. Each year, nearly 500,000 new cases of cervical cancer are diagnosed and over 270,000 women die from this disease around the world. Importantly, most of the cases (∼86%) and deaths (88%) occur in developing countries 1. Collectively, cervical cancer and the other HPV-associated malignancies represent 5.2% of all tumors affecting humans 11.

## DNA damage repair pathways and the HPV cycle

DNA contains the essential information for the formation and function of an organism, and its preservation is critical for survival. DNA is a fairly stable macromolecule; however, it suffers constant attacks from extracellular and intracellular factors that jeopardize its integrity. These factors include chemical and physical agents, infectious agents, highly reactive metabolic byproducts, and replication errors 12. In fact, DNA damage is a common event in human cells. It is estimated that each human cell suffers approximately one million lesions in its DNA every day 13. Therefore, if DNA damage is not efficiently repaired, the integrity of the genome is compromised, causing the accumulation of mutations that ultimately may lead to premature aging, cancer development and death 14,15.

DNA repair is the process by which alterations in DNA are reverted. Usually, this involves the action of nucleases, helicases, ligases and DNA polymerases. Different specialized DNA repair mechanisms exist in which complex arrays of cellular proteins detect and repair different kinds of DNA lesions 16. These mechanisms include base excision repair (BER), nucleotide excision repair (NER), DNA mismatch repair (MMR), homologous recombination repair (HRR) and nonhomologous end joining (NHEJ). Hereditary or acquired defects in any of these pathways may lead to genome instability with severe consequences for the individual 17.

The DNA damage response (DDR) is composed of different signal transduction pathways that include damage sensors, signal transducers and effectors that act in a coordinated fashion to maintain genome integrity. Some DNA lesions activate specific sensors, such as the MRN complex (Mre11-RAD50-NSB1), replication protein A (RPA) or ATR-interacting protein (ATRIP), that trigger signaling pathways by the phosphorylation of apical protein kinases, such as ataxia telangiectasia mutated (ATM), ataxia telangiectasia and Rad3 related (ATR) and DNA-dependent protein kinase (DNA-PK). Different types of DNA lesions activate different kinases 18,19. For instance, double-stranded DNA breaks may activate DNA-PK or ATM. On the other hand, single-stranded breaks preferentially activate ATR. The signaling cascade triggered by ATM and ATR then activates downstream kinases CHK2 (checkpoint kinase 2) and CHK1 (checkpoint kinase 1), respectively. However, the ATR-CHK1 and ATM-CHK2 pathways are not completely independent since they exhibit a high degree of redundancy and overlap. These pathways may regulate the phosphatase Cdc25 and activate the transcription factor p53, leading to cyclin-dependent kinase (CDK) inhibition and blocking cell cycle progression by the activation of specific checkpoints. Consequently, DNA damage effectors may mediate cell cycle arrest to allow DNA repair or, when the amount of damage is too high, induce cell death 14,17.

Many viruses, especially those with oncogenic properties, express proteins that affect the cell cycle and DNA damage repair regulatory pathways. Several studies have shown that during genome amplification, HPV proteins interact with different components of the cellular DNA repair machinery to activate or downregulate the expression or activity of factors of the ATM and ATR pathways. In 2009, Moody and coworkers showed that ATM activation is required for the productive genomic replication of HPV31 but not for episomal maintenance 20. On the other hand, it was observed that CHK1 inhibition causes an important reduction in the number of HPV episomes in differentiated cells 21. In addition, the knockdown of DNA topoisomerase 2-binding protein 1 (TopBP1), a protein that acts upstream of ATR, suppresses HPV31 replication 22. In line with these observations, it has been reported that E2 protein from HPV16 interacts with TopBP1 and that this interaction improves E2-mediated viral transcription and replication 23. Moreover, ATM and ATR kinases are constitutively activated in HPV-positive keratinocytes, and ATR/CHK1 blockade is associated with the downregulation of HPV productive replication and the reduced expression of late genes 22.

**Figure 1:**
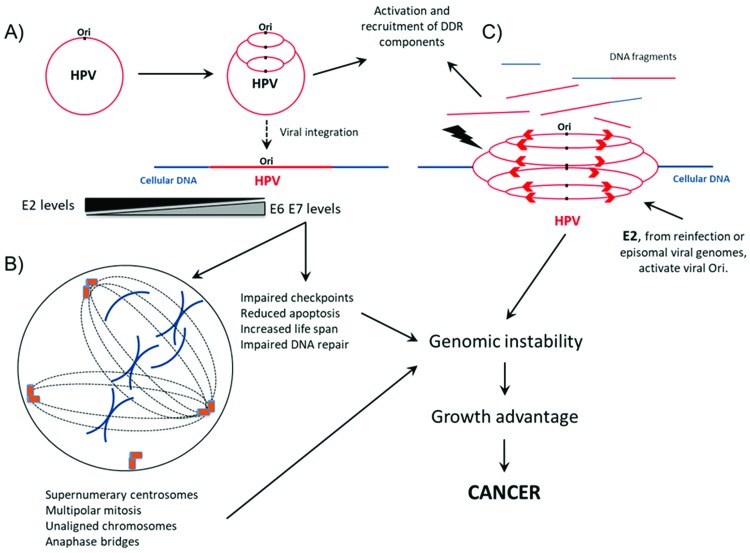
Interplay between HPV and cellular DNA repair machinery during infective viral cycle and virus-mediated cell transformation. A) During productive infection, HPV origin of replication (Ori) is triggered several times during the same cell cycle, leading to the “onion skin” type of DNA replication. To mediate genome amplification and process the viral genome replication intermediates, HPV activates and recruits several components of the DNA damage response (DDR), including 53BP1, ATRIP, OPbp1, p-ATM, p-H2AX, p-p53, ATR, CHK1, CHK2, PCNA, RPA, NBS1, BRCA1, RAD51, MRE11 and Ku70/80. HPV integration into the cellular genome may arise as a consequence of active DNA break repair. This event leads to E2 downregulation and increases the levels of E6 and E7 transcripts and proteins. B) In parallel to viral genome amplification, HPV oncogene expression increases the cell life span, induces alterations in chromosome segregation, prevents apoptosis and affects DNA repair, leading to the accumulation of alterations in cellular DNA. C) The presence of E2 protein either expressed from coexisting episomal viral genomes or derived from reinfection with the same or a different HPV type may trigger the replication of integrated HPV genomes, causing the rapid appearance of chromosome breaks and rearrangements, further contributing to malignant transformation. For references, see the main text.

Importantly, many proteins from ATM and ATR pathways colocalize with HPV replication sites, further supporting the role of these factors in the HPV life cycle. Different studies have shown that E1 and E2 early proteins may activate the DDR, recruiting factors involved in DNA repair to process replication intermediates during viral genome amplification. Identified proteins at centers of HPV replication include 53BP1, ATRIP, OPbp1, p-ATM, p-H2AX, p-p53, ATR, CHK1, CHK2, PCNA, RPA, NBS1, BRCA1, RAD51, MRE11 and Ku70/80 24-27 (Figure 1A). Collectively, these studies indicate that HPV may modulate different signaling pathways involved in the DDR to warrant effective viral transcription, faithful genome replication and, ultimately, the production of a large number of infective virions 10,28,29.

## HPV oncoproteins pave the road to genome instability

The effect of specific HPV proteins on DNA repair pathways has been addressed by different groups. The HPV genome does not express proteins capable of mediating viral DNA replication and depends on the host cell machinery to achieve genome amplification. Therefore, it must induce the host cell to re-enter the S phase of the cell cycle to obtain access to the DNA replication machinery 30. This is accomplished by the action of viral proteins, mainly E6 and E7, on cellular factors involved in cell cycle regulation. E6 and E7 are the major HPV transforming proteins, and their sustained expression is required to maintain the oncogenic properties of HPV-transformed cells in most scenarios 31-33. These proteins collaborate to immortalize primary cells and can independently abrogate the mitotic checkpoint and p53-mediated cell cycle arrest in response to DNA damage 34,35.

Among the main targets of E6 is the tumor suppressor protein p53. E6 forms a ternary complex with E6-associated protein (E6AP) and p53, altering p53's functional capacity and inducing the degradation of this cellular protein by the ubiquitin-mediated proteolysis pathway 36-38. In addition, E6 from HPV16 binds the transcriptional coactivator CREB-binding protein/p300 (CBP/p300) and downregulates its ability to activate p53-responsive elements in the promoters of several p53-regulated genes 39. E6 protein also upregulates the expression of the catalytic subunit of telomerase (hTERT) in primary cells and delays cell senescence 40. Moreover, E6 targets other cellular factors, including those regulating cell polarity (PDZ proteins), apoptosis and cell differentiation 40,41. Similarly, E7 from high-risk HPV types binds and induces the degradation of members of the retinoblastoma (pRb) tumor suppressor proteins pRb1^p110^, p107 and p130 (pRb2). This interrupts the interaction of pRb proteins with members of the E2F transcription factor family. Once free from inhibition, E2F activates the transcription of several genes involved in S phase induction and progression, including cyclins A and E 42,43. E7 also promotes cell cycle progression by downregulating the activity of cyclin-dependent kinase inhibitors (CKIs) p21 and p27 44,45. In addition, E7 of high-risk HPV16 and HPV31 interact with histone deacetylases 1 and 2 (HDAC1 and HDAC2), affecting the gene expression pattern of infected cells. Together, these data show that E6 and E7 affect the proliferation and differentiation of human keratinocytes by extending the lifespan of these cells 46,47.

Due to their impact on different cellular pathways, HPV oncoproteins may also promote the accumulation of genomic alterations that contribute to malignant transformation 48-54. For instance, Song and coworkers 55 demonstrated that mouse cells expressing E6 and E7 from high-risk HPV types continue to replicate DNA even in the presence of lesions induced by ionizing radiation (IR) and accumulate large numbers of alterations in the molecule. Similarly, HPV16 E7 expression is associated with the persistence of γH2AX and Rad51 foci upon the exposure of head and neck cancer cells to IR 56. Importantly, the expression of high-risk E6 and E7 proteins has been associated with the induction of DNA breaks and a high frequency of foreign DNA integration into host cells 35,57.

Several studies have shown that ATM and ATR pathways are targeted by HPV oncoproteins. For instance, Banerjee et al. 30 observed that the oncoprotein E7 from HPV18 induces increased levels of phosphorylated ATM and the downstream kinases CHK1, CHK2, and JNKs (c-Jun N-terminal kinases). It was also reported that E7 from HPV31 binds ATM, inducing its phosphorylation and activating CHK2 20. Another study showed that E7 from HPV16 induces the degradation of claspin, a protein from the ATR-CHK1 pathway, attenuating the DNA damage checkpoint 58. In addition, the results from recent studies support the involvement of HPV in skin cancer. In this context, it was observed that the protein E6 from HPV5 and HPV8, two important cutaneous HPV types, reduces ATM levels and downregulates the p300/ATR signaling axis, leading to the persistence of DNA lesions induced by UVB light 10,59. Finally, it was observed that E6 and E7 from HPV16 interact with breast cancer-associated protein 1 (BRCA1), inactivating its function in the repair of double-stranded DNA breaks 60.

Other alterations in DNA repair systems associated with HPVs have been described by different groups. One report has shown that oral keratinocytes immortalized with HPV16 exhibit deficiencies in the NER system and, consequently, are unable to remove cyclobutane pyrimidine dimers (CPDs) induced by UV light 61. A similar observation was made in fibroblasts expressing E7 from HPV16. These cells show defective NER activity and display a marked delay in the removal of CPDs induced by UVB light 62. Recently, it was observed that cells derived from squamous carcinoma of the head and neck positive for HPV are more sensitive to radiotherapy than HPV-negative cell lines due to a defect in BER in the the former cells 63. This finding is also in agreement with observations showing that E7 delays the repair of DNA lesions induced by IR in culture systems and laboratory animal models 56.

The oncoprotein E6 also targets DNA repair pathways. For instance, epithelial mammary cells expressing this protein from HPV16 exhibit a reduced capacity for removing thymine dimers after exposure to UV light 64,65. In addition, oral fibroblasts expressing E6 from HPV16 exhibit an impaired ability to repair double-stranded breaks by NHEJ. Interestingly, E6 achieves this effect via either p53-dependent or p53-independent pathways 66. Moreover, it has been described that E6 mediates O^6^-methylguanine DNA methyltransferase (MGMT) degradation via the ubiquitin/proteasome pathway in a process that requires the interaction of E6 with E6AP. The action of MGMT, which is impaired by E6, protects cells and tissues against the effects of alkylating agents 67. Finally, it was reported that E6 from different HPV types targets X-ray repair cross-complementing protein 1 (XRCC1) and inhibits its ability to repair DNA lesions 68.

These observations show that HPV infection may increase the frequency of DNA alterations in the host cell and delay the removal of these alterations by targeting DNA repair pathways. In addition, by impairing cell cycle checkpoints and apoptosis, HPV oncoproteins cause sustained proliferation while preventing cell death. Collectively, these effects may lead to cellular alterations that give rise to precursor lesions with a tendency for malignant progression.

## HPV-mediated genome instability and cancer

During tumor evolution, cells acquire genetic alterations that may upregulate the expression of proto-oncogenes or induce gain-of-function mutations in these genes. In addition, mutations, deletions and alterations in the methylation pattern of promoter sequences may downregulate the activity of tumor suppressor genes. Cells harboring these alterations may have proliferative advantages and acquire other phenotypical alterations, such as the capacity to invade other tissues and organs 69. Genome instability is a defining phenotype of most malignant tumors that comprises numerical and structural chromosomal abnormalities, as well as microdeletions, small insertions, the duplication of short nucleotide stretches and the accumulation of point mutations. Alterations in the sequences or expression level of proteins involved in DNA damage repair may result in the accumulation of genetic modifications important for cancer development. Normal cells exhibit an impressive array of mechanisms to detect and repair DNA defects. However, many of these mechanisms are altered in tumors cells 70,71. Although HPV activates several DNA repair pathways to assist its genome replication, as described above, HPV-induced tumors exhibit a high degree of genome instability. This fact seems to be critical for HPV-mediated carcinogenesis and suggests that DNA repair is attenuated during the steps leading from cellular transformation to cancer onset 7,35.

In hereditary cancers, mutations transmitted from progenitors predispose the host to the development of certain types of tumors and/or increase their sensitivity to carcinogens. This process may involve alterations in components of different DNA repair pathways. Several studies have suggested that mutations in this group of genes may act as cofactors in the establishment and progression of HPV-associated tumors. For instance, patients with Fanconi anemia (FA) or individuals carrying mutations in the breast cancer-associated (BRCA) gene exhibit a higher risk of developing HPV-associated cancerous lesions 72. In fact, the expression of HPV16 E7 in FA-deficient fibroblasts is associated with an increased number of chromosome aberrations 73. Moreover, K14E7/FancD2(-/-) mice exhibit a significantly higher incidence of head and neck squamous cell carcinoma than animals expressing K14E7 on a normal FancD2 background (+/+) 74. Using a similar approach, it was observed in FA-deficient mice that cervical tumors persisted even in the absence of HPV16 E7 expression, supporting the notion that FA-deficient tumors may escape from their dependency on the viral oncogene 32. Upregulation of the FA pathway is a frequent event in cervical SCC. *In vitro* data indicate that the activation of this pathway is mediated by E7 and is characterized by the formation of large FANCD2 foci and the recruitment of FANCD2 and FANCD1/BRCA2 to chromatin 73. These observations suggest that FA pathway activation plays a role in the HPV cycle. The malfunction of this pathway may lead to the accumulation of HPV-mediated genomic alterations and the promotion of tumor development in FA patients. Of clinical relevance, in this context, where FA facilitates the accumulation of mutations, sustained viral oncogene expression may no longer be required to maintain the transformed phenotype 32.

Alterations in the expression of genes involved in DNA damage sensing and repair are readily detected in HPV-associated cancers and precursor lesions. A study that analyzed the expression of genes involved in BER in 50 invasive cervical cancer patients and 40 squamous intraepithelial lesions (SILs) showed that the expression of *XRCC1, ERCC2, ERCC4* and *ERCC1*, at both the mRNA and protein levels, was downregulated in tumors and precursor lesions compared to samples from control subjects 75. Recently, Seiwert et al. 76 sequenced 617 cancer-associated genes in 120 matched head and neck squamous cell carcinoma/normal samples, of which 42.5% were positive for HPV DNA. They observed that HPV-positive tumors showed 5.8% of DNA repair gene aberrations (including 7.8% of BRCA1/2 mutations). In a study conducted in our laboratory, we compared the expression pattern of 135 genes involved in DNA damage repair/signaling between normal human keratinocytes and cervical cancer-derived cell lines and observed that the mRNA levels of 18 genes are altered in HPV-transformed cell lines 77.

Genome instability, in the context of HPV infection, may arise through different molecular pathways. A clear example of this is represented by the results of a study conducted by Kadaja et al. 25. The authors reported that the ectopic expression of HPV18 E1 and E2 triggers HPV replication from viral-integrated HPV genomes in SiHa and HeLa cells, leading to the accumulation of chromosome defects. Different from the cellular origin of replication, which is activated once per cell cycle, the viral origin of replication can be triggered several times during the same cell cycle, leading to the “onion skin” type of DNA replication. This process generates replication intermediates that stress the DNA molecules, leading to double- or single-stranded breaks and the recruitment of DNA repair machinery that ligates loose DNA ends and promotes chromosomal rearrangement. These observations raise the disturbing possibility that the coexistence of integrated and episomal HPV genomes in the same cell may induce chromosome aberrations arising from the integrated viral DNA. Moreover, the de novo HPV infection of cells harboring integrated DNA genomes may also endanger the cellular genome integrity and favor cell transformation (Figure 1C).

Chronic inflammation also plays a major role in cancer biology 69. This is also true for tumors associated with HPV infection 78. For instance, cells harboring HPV16 genomes exhibit increased levels of nitric oxide (NO), which triggers inflammation and promotes DNA breaks 79. In addition, extracellular factors, mainly inflammatory mediators, may affect HPV infection outcome. For instance, it has been observed that interferon β may induce the elimination of HPV16 episomes from naturally infected cervical keratinocytes by selecting cells with integrated viral genomes 80. In addition, interferon may induce HPV genome integration 81. These observations suggest that chronic inflammation may potentiate viral persistence and, consequently, lesion establishment and progression 82. Importantly, these findings indicate that HPV therapies should be evaluated for the potential selection of cells with integrated genomes and increased proliferative potential.

As previously stated, HPV oncogenes E6 and E7 play a critical role in HPV-mediated carcinogenesis by affecting major cellular processes. The consequences of HPV oncoprotein actions are reflected on cellular genome homeostasis. For instance, inhibition of the postmitotic checkpoint by high-risk HPV E6 and E7 is associated with the induction of polyploidy in human keratinocytes 83-85. It was also observed that HPV16 oncoprotein expression induce supernumerary centrosomes and multipolar mitotic spindles that may lead to aneuploidy 49. In addition, an independent study showed that E6 oncoprotein causes centrosome accumulation, while HPV16 E7 interferes with the centrosome duplication cycle 86. Interestingly, while E7 from HPV16 may induce the delocalization of dynein from mitotic spindles, this has not been correlated with mitotic defects 87 (Figure 1B).

Alterations in the regulation of the cell cycle and the inhibition of proapoptotic factors are important underlying events in HPV-mediated genome instability. As expected, genomic instability is an early event during HPV infection that precedes viral integration, the development of associated lesions and their eventual progression to cancer 88-90. Genomic instability is crucial in the appearance of aneuploid cells and lesion progression 88. This is highlighted by the fact that highly polyploid as well as aneuploid cells are mainly detected in high-grade cervical lesions 91. Of note, most of the alterations described are restricted to cells infected with high-risk HPV types and are not detected upon infection with low-risk types 92. Studies conducted using clinical samples have shown that cervical tumors exhibit a plethora of chromosomal alterations, including gains in 1, 3q, 5p, 6p, 7, 8q, 9q, 16q, and 20 and losses in 2q, 3p, 4q, 6q, 11q, 13q, 16, and 17 93-98. Finally, genomic alterations associated with HPV infection have also been observed in tumors from different anatomical locations, including oral, anal, laryngeal and head and neck neoplasias 76,99-101.

Genome instability is probably not a consequence of HPV-mediated malignant transformation. However, it may favor the acquisition of genetic alterations that confer growing advantages to cells expressing viral oncogenes. In addition, the underlying mutator phenotype may allow transformed cells to adapt rapidly to the harsh conditions of the tumor microenvironment, contributing to tumor onset and progression.

## HPV-associated disease treatment: can we target DNA repair systems?

The data discussed above further support the established notion that the main mechanism by which HPV induces cell transformation is the targeting of p53 and pRb by E6 and E7, respectively 43,102-104. Therefore, it is assumed that the downregulation of these tumor suppressor proteins by E6 and E7 mimics the effect of inactivating mutations observed in p53 and pRb in tumors not associated with HPV infection. Consequently, it is believed that HPV-positive cancers, including anogenital, oropharyngeal tract and anal canal tumors, are less likely to present p53 and pRb mutations 105,106. Conversely, different studies have shown that HPV-transformed cells retain the ability to respond to genotoxic stress by inducing a p53-mediated response. Therefore, p53 downregulation as a consequence of HPV oncogene action is not functionally equivalent to p53 inactivation by mutation 107-109. This may be at least one of the underlying molecular mechanisms explaining why the presence of HPV is associated with a better response to therapy and constitutes a positive prognostic factor for patients with oropharyngeal tumors 110.

However, the implementation of new therapeutic strategies to treat HPV-associated tumors is still required. In this context, the dependence of HPV-transformed cells on cellular DNA repair machinery may constitute a suitable target for intervention. In fact, inhibitors of selected molecules involved in specific cellular pathways have been applied for the treatment of cervical cancer in clinical trials 111,112. In addition, several examples of synthetic lethality involving genes from DNA damage repair systems and tumor suppressor-regulated pathways have been described and suggested as potential targets for cancer therapy 113.

The first case of synthetic lethality involving genes associated with DNA damage repair was with poly(ADP-ribose) polymerase (PARP) and breast and ovarian cancer susceptibility genes BRCA1 and BRCA2 114,115. PARP is a family of proteins involved in several cellular processes, including DNA repair and the maintenance of genome stability. PARP1, the most studied member of the family, is rapidly recruited to nicks and double-stranded breaks in cellular DNA, where it gathers components of the DNA repair machinery and promotes the removal of lesions 116. In fact, cells with alterations in different DNA damage repair pathways exhibit increased susceptibility to the loss of PARP activity. As expected, PARP inhibitors (iPARPs) prevent DNA damage repair and are used in cancer therapy, particularly in tumors with germline or somatic mutations in BRCA1/2 117. The administration of iPARPs promotes cell death by downregulating BER and promoting the accumulation of DNA defects in the cell 116,118-121. Additionally, by preventing DNA repair, PARP inhibition increases the sensitivity of cells to chemotherapeutical agents that promote DNA lesions 122.

E6 and E7 form high-risk HPV types are pleotropic proteins that target an increasing list of cellular factors affecting their expression and function. As such, HPV-transformed cells exhibit major alterations in important signaling pathways and cellular processes, including several DNA damage repair mechanisms. This fact has consequences of clinical relevance. For instance, patients harboring HPV-associated head and neck squamous cell carcinoma (HNSCC) have significantly improved survival compared with patients affected by HPV-negative tumors. Although the molecular mechanisms underlying this difference are not completely understood, it is accepted that impaired DNA repair abilities, probably due to HPV oncoproteins action, play a major role 110,123. In fact, HPV-positive HNSCC-derived cell lines accumulate more double-stranded DNA breaks than HPV-negative counterparts and exhibit higher radiosensitivity 63,124. In conclusion, HPV-transformed cells exhibit major defects in DNA repair. Nevertheless, we anticipate that HPV-transformed cells depend on the preservation of a basal level of DNA repair activity meditated by cellular machinery to maintain the minimal genomic stability needed for tumor cell viability. Therefore, a great effort should be directed toward identification of the molecular pathways necessary for the survival HPV-driven tumor cells. This will certainly contribute to the development of more efficient antitumor therapies.

## AUTHOR CONTRIBUTIONS

Prati B prepared the text describing the effect of HPV on DNA repair machinery. Marangoni B prepared the text describing HPV-associated disease treatment strategies. Boccardo E prepared, revised and corrected all the text.
